# Predicting total knee arthroplasty from ultrasonography using machine learning

**DOI:** 10.1016/j.ocarto.2022.100319

**Published:** 2022-11-06

**Authors:** Aleksei Tiulpin, Simo Saarakkala, Alexander Mathiessen, Hilde Berner Hammer, Ove Furnes, Lars Nordsletten, Martin Englund, Karin Magnusson

**Affiliations:** a Research Unit of Medical Imaging, Physics and Technology, University of Oulu, Oulu, Finland; b Department of Diagnostic Radiology, Oulu University Hospital, Oulu, Finland; c Center for treatment of Rheumatic and Musculoskeletal Diseases (REMEDY), Diakonhjemmet Hospital, Oslo, Norway; d Faculty of Medicine, University of Oslo, Oslo, Norway; e The Norwegian Arthroplasty Register, Department of Orthopaedic Surgery, Haukeland University Hospital, Bergen, Norway; f Department of Clinical Medicine, University of Bergen, Bergen, Norway; g Division of Orthopaedic Surgery, Oslo University Hospital, Oslo, Norway; h Clinical Epidemiology Unit, Orthopaedics, Department of Clinical Sciences Lund, Faculty of Medicine, Lund University, Lund, Sweden; i Norwegian Institute of Public Health, Cluster for Health Services Research, Oslo, Norway

**Keywords:** Total knee replacement, Ultrasonography, Machine learning, Multivariate predictive modeling

## Abstract

**Objective:**

To investigate the value of ultrasonographic data in predicting total knee replacement (TKR).

**Design:**

Data from the Musculoskeletal Pain in Ullensaker study (MUST) was linked to the Norwegian Arthroplasty Register to form a 5–7 year prospective cohort study of 630 persons (69% women, mean (SD) age 64 (8.7) years). We examined the predictive power of ultrasound (US) features, i.e. osteophytes, meniscal extrusion, synovitis in the suprapatellar recess, femoral cartilage thickness, and quality for future knee osteoarthritis (OA) surgery. We investigated 4 main settings for multivariate predictive modeling: 1) clinical predictors (age, sex, body mass index, knee injury, familial OA and workload), 2) radiographic data (assessed by the Kellgren Lawrence grade, KL) with clinical predictors, 3) US features and clinical predictors. Finally, we also considered an ensemble of models 2) and 3) and used it as our fifth model. All models were compared using the Average Precision (AP) and the Area Under Receiver Operating Characteristic Curve (AUC) metrics.

**Results:**

Clinical predictors yielded AP of 0.11 (95% confidence interval [CI] 0.05–0.23) and AUC of 0.69 (0.58–0.79). Clinical predictors with KL grade yielded AP of 0.20 (0.12–0.33) and AUC of 0.81 (0.67–0.90). The clinical variables with ultrasound yielded AP of 0.17 (0.08–0.30) and AUC of 0.79 (0.69–0.86).

**Conclusion:**

Ultrasonographic examination of the knee may provide added value to basic clinical and demographic descriptors when predicting TKR. While it does not achieve the same predictive performance as radiography, it can provide additional value to the radiographic examination.

## 1 Introduction

The rising incidence of total knee replacement (TKR) surgery during the past decades is a growing concern across the world [[1], [2], [3], [4], [5]]. These surgeries are performed primarily due to osteoarthritis (OA), and most patients report good clinical outcomes [6]. However, the costs of these procedures are high and exceed $10 billion annually in the United States [7]. Furthermore, TKR may require revisions, which alone reach $2.7 billion in hospital charges in the United States [8] and have a significant impact on the quality of life of patients with OA.

In recent decades, there has been a growing interest in developing predictive models for TKR [[9], [10], [11], [12]] and OA progression in general [13,14]. If persons at high risk of TKR or OA progression could be identified at early stages, behavioral interventions such as weight loss and exercise programs may be implemented to prevent further rapid development of the disease [15]. Further, prediction models may be used for a more accurate selection of individuals into clinical trials or observational studies. Most of the previous works show two main components needed to obtain fairly good predictive performance: 1) the use of imaging data and 2) the use of advanced modeling methods, based on machine learning (ML). The main benefit of ML, compared to statistical inference, is the objective to optimize a metric of interest, such as the performance on unseen (test) data.

The major clinical limitation of previous development of predictive models is the use of modalities that are costly, such as magnetic resonance imaging (MRI). Although contributing to the understanding of the etiology of OA, they are less useful in the clinical setting when the aim is to predict OA progression. As an example, for suspected early OA in the clinical setting, there is a need for more available modalities that provide the diagnostic and predictive value at least comparable to the existing ones.

Although ultrasonography (US) requires thorough expert training and is currently not a part of the standard clinical evaluation of OA, it is a promising future imaging tool as it uniquely allows for the immediate assessment of soft tissues, such as menisci and cartilage [16]. In contrast to X-ray, it enables a three-dimensional assessment of the joint without emitting radiation. Recent works indicate that US has a potential to identify OA features almost to an equal extent as MRI [17], and to our knowledge, no studies have assessed its value in predicting TKR. We hypothesized that when used with advanced modeling methods, US could serve as a low-cost and easily available tool to predict TKR, and we aimed to investigate it in the present work.

## 2 Methods

### 2.1 Data

#### 2.1.1 The MUST study

We used data from the Musculoskeletal Pain in Ullensaker study (MUST), a population-based prospective cohort study in South-Eastern Norway that was initiated in 2010 and linked to the Norwegian Arthroplasty Register in 2017 (approved by Regional Ethics Committee). In total, 630 persons with knee, hip and/or hand OA as reported on postal questionnaires attended an extensive baseline clinical examination in 2010–12 [18]. Other than having complete data on knee ultrasound and radiographs and no knee prosthesis surgery at the joint level at baseline, we had no specific inclusion/exclusion criteria. Participants were allowed to have knee pain at baseline but were not required to. The Norwegian Arthroplasty Register covers >95% of all prosthesis surgeries in Norway with registration of cause and date of surgery as well as joint site [19]. More than 80% of TKRs in Norway are done for primary OA [20]. Incident arthroplasty due to primary OA in the left or right knee joint were our main outcome variables. Thus, arthroplasty due to other causes than OA (i.e. fractures, inflammatory rheumatic diseases etc.) were excluded.

#### 2.1.2 Ultrasonography

A sonographic examination of both knee joints of all participants was performed at baseline using the same ultrasound machine across all examinations (Siemens Medical Solutions, Excellence version, Mountain View, California, USA), with fixed settings used for all knees (a 5–13 ​MHz linear array transducer, power Doppler with frequency 7.3 ​MHz and pulse repetitive frequency 391 ​Hz). Two sonographers (a rheumatologist with 15+ years of ultrasound experience (HBH) and a trained medical student with 2+ years of experience (AM)) performed semiquantitative assessments together and reached consensus on each joint scoring, making it possible to discuss the grades consecutively if disagreement. The sonographers were blinded to clinical and other imaging results. Femoral and tibial osteophytes were scored for the lateral and medial side on a 0–3 scale (0 ​= ​none, 1 ​= ​minor, 2 ​= ​moderate and 3 ​= ​major size of osteophytes) with the participant lying in supine position with knees extended. In the same position, medial and lateral meniscal protrusions were scored 0–2 (0 ​= ​none, 1 ​= ​minor and 2 ​= ​major) whereas synovitis in the suprapatellar recess was assessed as a combined evaluation of effusion and synovitis scored 0–3 (0 ​= ​none, 1 ​= ​minor, 2 ​= ​moderate, 3 ​= ​major). With the knee maximally flexed, femoral cartilage thickness (FCT) was assessed in millimeters for the medial and lateral condyle and the sulcus. The femoral cartilage quality (FCQ) was scored 0–2, i.e. from normal to considerable hyperechoic/absent (0 ​= ​normal, 1 ​= ​minor, 2 ​= ​major). Femoral hyaline cartilage was scored as present or absent of calcium crystal pyrophosphate depositions (none vs present). Examples of ultrasound images, their scoring as well as assessment of intra- and interrater reliability are provided in our previous work [21].

#### 2.1.3 X-ray imaging

Knee X-rays were obtained at the baseline examination using a positioning frame (SynaFlexer™) with standardised knee flexion angle to 20° and external foot rotation to 5° (10° beam angle) by a medical student. The x-rays views include anterior-posterior, lateral and patella tangential. The radiographs were scored by a medical student from 0 to 4 according to the Kellgren-Lawrence (KL) atlas [22] using the software Pacs in 2012–13. KL grade 0 indicates no OA, 1 – doubtful OA, 2 – early OA, 3 – moderate OA, and KL 4 – severe OA.

#### 2.1.4 Clinical variables

In addition to the joint imaging data, we included predictors from the most widely validated and most widely used prediction model in knee OA to date [9,23]. Body mass index (BMI), kg/m^2^ was calculated from measured height and weight. Information on up to three knee injuries per knee was validated by a nurse and grouped into present or absent knee injury (ever in life) until the date of examination. Familial OA was self-reported as any OA present in mother, father and/or sibling. Occupational load was self-reported as having any current or previous work activities including heavy lifting, work in challenging positions etc.

### 2.2 Modeling approach

#### 2.2.1 Logistic regression

Logistic regression (LR) is a parametric linear model, which predicts the probability of a binary outcome. In the field of OA, LR has been widely used for TKR and other prediction tasks [1,9,13,[24], [25], [26]], and we therefore chose it as our reference modeling method. Conventionally in medical literature, LR is fit to data, and its coefficients are later looked at to understand the relation between the covariates and the outcomes. In the case of our work, however, we target the accuracy of prediction using highly correlated covariates. To account for this, we used $L_{2}$ regularization technique (also known as Ridge penalty) [27,28], and searched for its strength, using the cross-validation (CV) procedure defined in the sequel.

#### 2.2.2 Model configurations

Due to the expected sparsity of ultrasound findings for osteophytes and cartilage quality when these were assessed on an ordinal scale (0–3 and 0–2, respectively), we created new variables for inclusion in our models. Firstly, we derived an osteophytes binary variable, which was indicative of any osteophytes with grade ≥1 detected by an assessor in ultrasound. We also created a variable that was indicative of the cartilage quality. Here, we collapsed the presence of hyalin cartilage and femoral cartilage quality grade ≥1 into a single variable (from now called femoral cartilage quality).

In the multivariate analyses, we evaluated the predictive ability of five different multivariate models including different variations of clinical characteristics, KL grade and ultrasound features as described in Table 1. Our final approach for prediction (model 5) was based on a combination of two predictive models – the one with radiography, and the one with ultrasound, with both including clinical data. Ensembling of different models helps to increase model performance and can be viewed as a voting of two experts with different background. In our study, we averaged two probability outputs coming from models 2 and 3 to obtain model 5 [29].

**Table 1:** Evaluated model configurations.

Model	Features included to the model
Model 1	Age, Sex, BMI, knee injury, familial OA, occupational load
Model 2	Model 1 features ​+ ​KL grade
Model 3	Model 1 features ​+ ​ultrasound features
Model 4	Model 1 features ​+ ​ultrasound features ​+ ​KL grade
Ensemble	Ensemble of models 2 and 3

BMI=Body Mass Index.

OA=Osteoarthrtis

KL grade ​= ​Kellgeren Lawrence grade.

### 2.3 Experimental setup

#### 2.3.1 Nested leave-one-out cross-validation

**Fig. 1:**
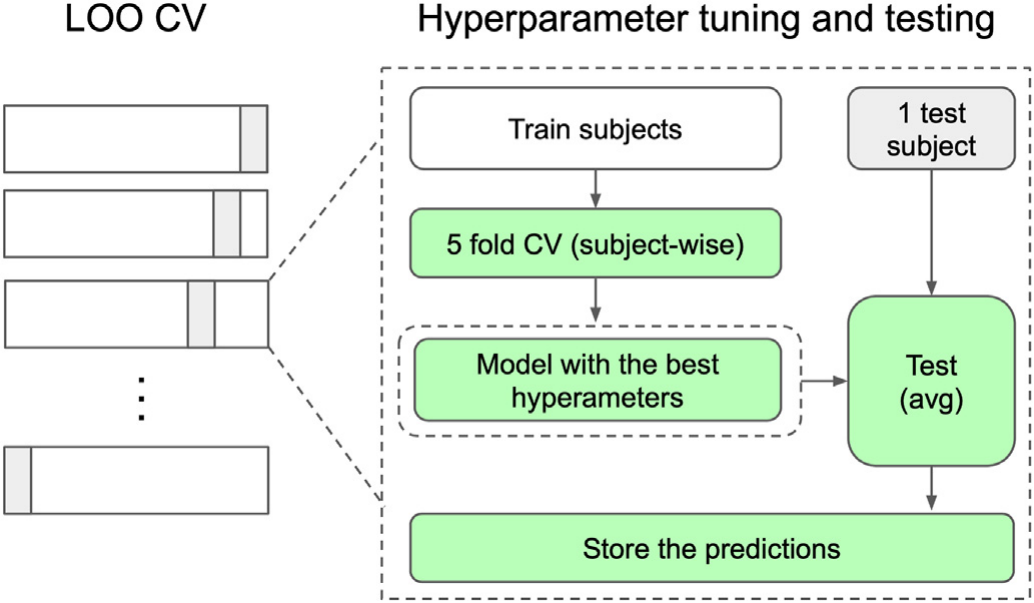
Nested LOO-CV (leave-one-patient-out cross-validation) procedure. In the outer loop of our pipeline, we removed one subject (one or two knees) from the training data. On the remaining part, we searched for optimal hyperparameters, test on the left-out subject using 5 models, corresponding to the best set of hyperparameters from all the folds, and store the results. This process is repeated for all subjects in the dataset.

To assess the generalization of our modeling approach, we used a nested cross-validation procedure. As our dataset has multiple measurements from the same patient, leave-one-patient out cross-validation (LOO-CV) strategy was applied [30]. This was done to mitigate bias in estimating cross-validation (CV) error, which comes from overfitting to the best set of hyperparameters. For each such a split, we searched for the model hyperparameters using another, nested 5-fold patient-wise cross-validation loop, which was individually created for every split. Using these splits, we also searched for models' thresholds to produce binary (dichotomized) predictions. Eventually, we retrained the model using the best hyperparameters found on the nested CV to make a prediction for the test subject, and then averaged the results. A graphical illustration of our CV pipeline is shown in Fig. 1.

#### 2.3.2 Analyses and experimental details

First, we studied the descriptive statistics of the dataset of all the included clinical characteristics and imaging features. We examined whether each of the ultrasound features, as well as the KL grade and clinical predictors, were predictive of knee OA surgery in the same joint in univariate analyses using logistic regression and computed the odds ratio for each of them. Similar to the univariate analyses, we used odds ratios as means to explain the models. However, as the results for the multivariate predictive models are evaluated using the LOO-CV, we get multiple estimates of the model parameters. Thus, in the paper, we report the mean and standard deviations of odds ratios across LOO-CV iterations. All analyses were performed on the joint level, meaning that the models did not need to take two knees from the same individual during training, but we made sure that two knees from the same patient were always either in the train or in the test sets in the internal CV loop.

To implement the logistic regression, we used scikit-learn v0.24.2 [31]. LOO-CV routine was implemented *ad-hoc*, and the computations were made parallel (per one testing subject) (Fig. 1). The regularization coefficient for the logistic regression was searched optimized using the internal CV loop. We used L-BFGS-B optimizer [32] for model training. After computing the LOO-CV, we were able to test the performances of all the models described in Table 1.

We used the area under the receiver operating characteristic (ROC-AUC) and the area under the precision-recall curve (AP). In classification problems, the ROC curves reflect the trade-off between the true positive rate and the true negative rates of a classifier. The precision-recall curve reflects the trade-off between the positive predictive value of the classifier and the true positive rate [33]. The area under the derived curve is then used as AP. The model performances were compared using the 1000 times stratified bootstrap with 95% CI.

## 3 Results

### 3.1 Descriptive findings

Of the in total 630 persons with available baseline data, 40 persons had already undergone total knee surgery, 29 persons had missing data on ultrasound and/or other features and 4 joints had surgery due to other reasons during follow-up. Thus, we excluded in total 73 persons and studied in total 557 persons with their 1114 knee joints. The study sample consisted of 69% women and with the age 64 ​± ​8.7 years old (Table 2). After 5–7 years (because some had their baseline examination in 2010 and others in 2012), in total 30 (2.7%) persons had an incident total knee surgery due to primary OA.

**Table 2:** Baseline characteristics of the MUST at the knee joint level.

	Knee joints without later surgery N ​= ​1084	Joints with later surgery N ​= ​30
**Clinical characteristics**		
Age, mean (SD)	63.8 (8.7)	63.7 (7.3)
Women, n (%)	748 (69.0)	21 (70.0)
Body Mass Index (kg/m^2^), mean (SD)	27.9 (4.6)	30.7 (5.1)
Knee injury, n (%)	201 (18.5)	15 (50.0)
Familial OA, n (%)	582 (53.7)	21 (70.0)
High occupational load, n (%)	228 (21.0)	7 (23.3)
Knee pain last week, n (%)	428 (39.5)	25 (83.3)
**Radiographic scoring**		
Kellgren Lawrence, grade 0, n (%)	726 (67.0)	5 (16.7)
Kellgren Lawrence, grade 1, n (%)	189 (17.4)	3 (10.0)
Kellgren Lawrence, grade ≥2, n (%)	169 (15.6)	22 (73.3)
**Ultrasonographic scoring**		
≥1 Osteophytes, n (%)	318 (29.6)	25 (86.2)
Medial or lateral meniscal extrusion grade ≥1, n (%)	279 (25.7)	5 (16.7)
Suprapatellar synovitis grade ≥1, n (%)	261 (24.1)	15 (50.0)
Femoral cartilage thickness, mm, mean (SD)		
Medial	2.1 (0.6)	2.5 (0.6)
Lateral	2.1 (0.6)	2.3 (0.5)
Sulcus	2.7 (0.8)	3.1 (0.7)
Femoral cartilage quality grade ≥1, n (%)	179 (16.5)	11 (36.7)
Femoral hyalin cartilage grade ≥1, n (%)	6 (0.6)	0 (0)

Mm; millimeter, kg; kilogram, m; meter, OA; osteoarthritis, SD; standard deviation.

### 3.2 Univariate analyses

We first conducted univariate analyses to assess the predictive power of individual covariates (Table 3). Based on these analyses, one can see that previous injury (OR of 4.39) and familial OA (OR of 2.01) are the most predictive of TKR among the patients’ characteristics. However, in the univariate setup, the presence of past injury yielded close to random performance – AP of 0.05 (0.03–0.07) and AUC of 0.66 (0.57–0.74) despite being significant. Familial OA yielded AP of 0.03 (0.03–0.04) and AUC of 0.58 (0.49–0.66) and was not significant. Radiographic information presented by a KL-grade had an OR of over 3 and was a significant predictor. It yielded AP of 0.18 (0.11–0.27) and AUC of 0.84 (0.74–0.91).

**Table 3:** Univariate analyses of some individual features with 95%confidence intervals (CI). CI were computed with stratified bootstrapping with 1000 iterations.

Data source	Feature	OR	P-value	AP	AUC
Patient	Age	1.00	0.98	0.03 (0.02–0.04)	0.48 (0.39–0.57)
	Sex	1.05	0.91	0.03 (0.02–0.03)	0.50 (0.42–0.58)
	Body Mass Index	1.12	0.00	0.06 (0.04–0.10)	0.67 (0.56–0.77)
	Injury	4.39	0.00	0.05 (0.03–0.07)	0.66 (0.57–0.74)
	Familial OA	2.01	0.08	0.03 (0.03–0.04)	0.58 (0.49–0.66)
	Occupational load	1.14	0.76	0.03 (0.03–0.04)	0.51 (0.44–0.59)
Radiography	KL-grade	3.13	0.00	0.18 (0.11–0.27)	0.84 (0.74–0.91)
Ultrasonography	OST	16.17	0.01	0.04 (0.03–0.04)	0.66 (0.61–0.69)
	Synovitis grade ≥1	2.06	0.00	0.05 (0.03–0.10)	0.64 (0.54–0.73)
	Meniscal extrusion grade ≥1	0.58	0.27	0.03 (0.03–0.03)	0.45 (0.39–0.52)
	Femoral cartilage thickness, mm				
	Lateral	1.60	0.14	0.04 (0.03–0.05)	0.60 (0.48–0.69)
	Sulcus	1.96	0.00	0.05 (0.03–0.08)	0.66 (0.55–0.74)
	Medial	2.65	0.00	0.06 (0.04–0.13)	0.66 (0.56–0.75)
	Femoral cartilage quality	2.81	0.01	0.04 (0.03–0.06)	0.60 (0.51–0.68)

OA ​= ​osteoarthritis.

OR ​= ​odds ratio.

AUC ​= ​Area under the ROC curve.

AP ​= ​Average Precision.

OST=Presence of osteophytes with grade ≥1

Among the ultrasound variables, the presence of osteophytes (OR of 16.17), synovitis (OR of 2.06), FCT in sulcus (OR of 1.96) and medial (OR of 2.65) compartments, and FCQ (OR of 2.81) were also found to be significant predictors (Table 3). All these predictors yielded AP over 0.03. FCT in the medial compartment yielded the highest AP among all other US features in univariate analyses - 0.06 (0.04–0.13). FCT in sulcus yielded AP of 0.05 (0.03–0.08), which was similar to the presence of synovitis – AP of 0.05 (0.03–0.10). AUC values for each of the covariates are reported in Table 3.

### 3.3 Multivariate predictive modeling

After executing the univariate analyses, we evaluated our machine learning approach on models shown in Table 1. These results are presented in Table 4. We found that the model containing the KL grade and the basic patient characteristics (model 2) yielded the best AP scores among the models 1–4: AP of 0.20 (0.12–0.33) and AUC of 0.81 (0.67–0.90). Model 3, which relied of patient characteristics and features from ultrasound examination yielded AP of 0.17 (0.08–0.30) and AUC of 0.79 (0.69–0.86).

**Table 4:** Multivariate modeling results with 95%confidence intervals (CI) on leave-one-subject-out cross-validation. For the performance metrics CIs, were used stratified bootstrapping with 1000 iterations. AP of 0.03 indicates random performance. N/A indicates that feature has not been included into the model. Features from synovitis to FCT-M were assessed from ultrasonographic examination. Ensemble of models 2 and 3, which averaged their output probabilities yielded AP of 0.24 (0.13–0.39) and AUC of 0.83 (0.73–0.91).

Feature importance				
Feature	Model 1	Model 2	Model 3	Model 4
Age	1.1 (1.11–1.11)	0.89 (0.88–0.89)	1.1 (1.08–1.09)	0.97 (0.97–0.97)
Sex	1.1 (1.09–1.10)	1.1 (1.09–1.10)	1.3 (1.27–1.28)	1.1 (1.14–1.16)
BMI	1.7 (1.67–1.68)	1.2 (1.16–1.16)	1.4 (1.44–1.45)	1.2 (1.16–1.17)
Injury	1.8 (1.81–1.83)	1.3 (1.26–1.26)	1.5 (1.45–1.46)	1.2 (1.18–1.18)
Familial OA	1.4 (1.43–1.44)	1.3 (1.29–1.30)	1.4 (1.38–1.39)	1.2 (1.19–1.20)
Occupational load	1.1 (1.08–1.09)	1.0 (1.05–1.05)	1.0 (1.03–1.03)	1.0 (1.01–1.01)
Synovitis grade	N/A	N/A	1.4 (1.39–1.39)	1.2 (1.19–1.19)
OST	N/A	N/A	2.0 (1.94–2.01)	1.2 (1.22–1.23)
ME	N/A	N/A	0.93 (0.93–0.93)	0.95 (0.95–0.95)
FCQ	N/A	N/A	1.3 (1.29–1.30)	1.1 (1.09–1.09)
FCT-L (lateral)	N/A	N/A	0.98 (0.98–0.98)	1.0 (1.01–1.02)
FCT-S (sulcus)	N/A	N/A	1.5 (1.51–1.52)	1.3 (1.28–1.30)
FCT-M	N/A	N/A	1.3 (1.29–1.29)	1.2 (1.15–1.16)
KL-Grade	N/A	2.5 (2.48–2.54)	N/A	1.9 (1.90–1.96)
**Performance**				
**Metric name**	**Model 1**	**Model 2**	**Model 3**	**Model 4**
AP	0.11 (0.05–0.23)	0.20 (0.12–0.33)	0.17 (0.08–0.30)	0.20 (0.12–0.34)
AUC	0.69 (0.58–0.79)	0.81 (0.67–0.90)	0.79 (0.69–0.86)	0.82 (0.71–0.90)

BMI=Body Mass Index.

OA=Osteoarthritis.

OST=Presence of osteophytes with grade ≥1.

ME ​= ​Meniscal extrusion.

FCQ=Femoral cartilage quality.

FCT-L ​= ​Femoral lateral cartilage thickness.

FCT-S=Femoral sulcus cartilage thickness.

FCT-M ​= ​Femoral medial cartilage thickness.

**Fig. 2:**
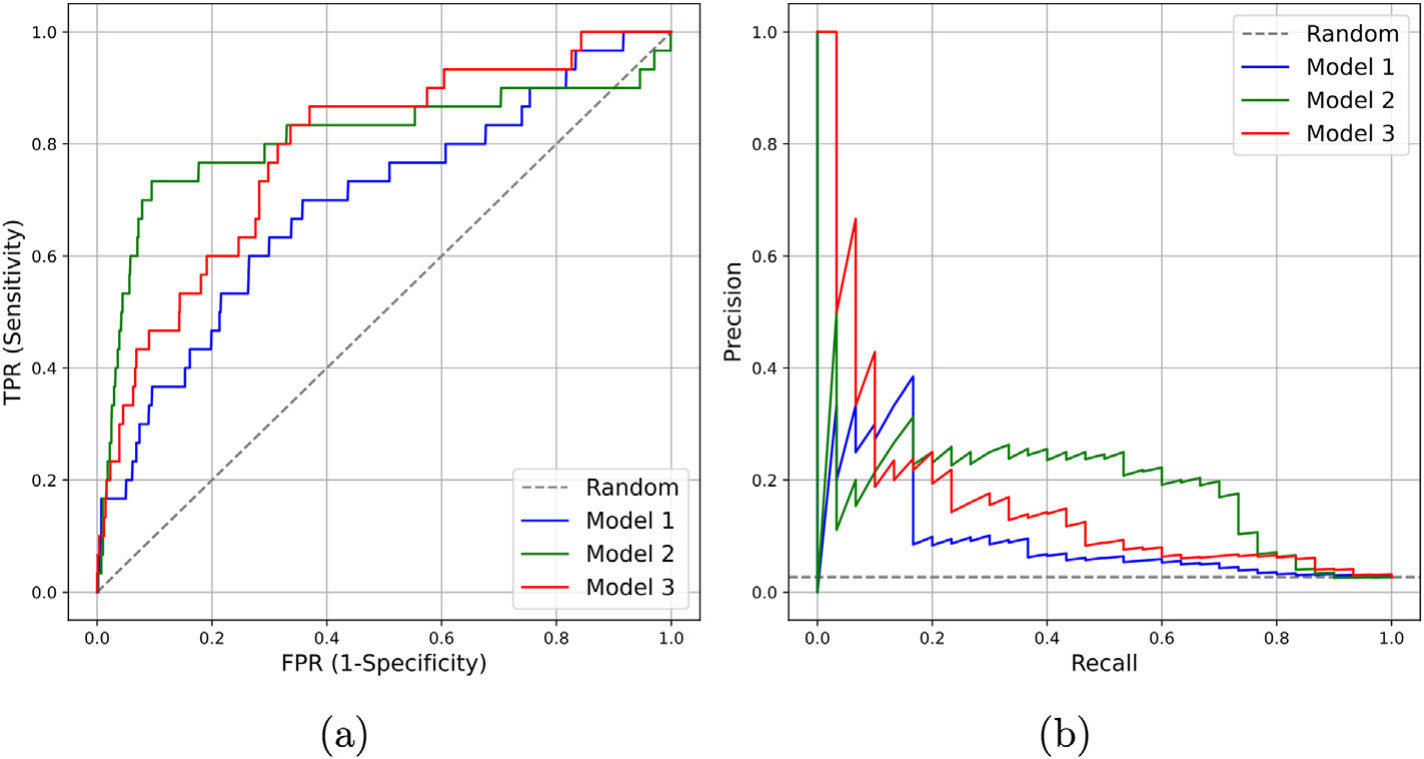
Receiver Operating Curve (ROC) and Average Precision (AP) curves for the base model, base model with ultrasonographic data, and base model with the Kellgren-Lawrence (KL) grade.

Fig. 2 shows ROC and PR curves for the base model (model 1), and two base models with imaging – the one with the KL grade (model 2), and the one with the ultrasound features (model 3). One can see that the imaging models behave differently in both ROC and AP spaces. Based on these findings, we ensembled the predictions of models 2 and 3, and compared the result to model 4, which naïvely incorporates the data from radiography and ultrasonography in a single model, without model ensembling (see all models features in Table 4).

**Fig. 3:**
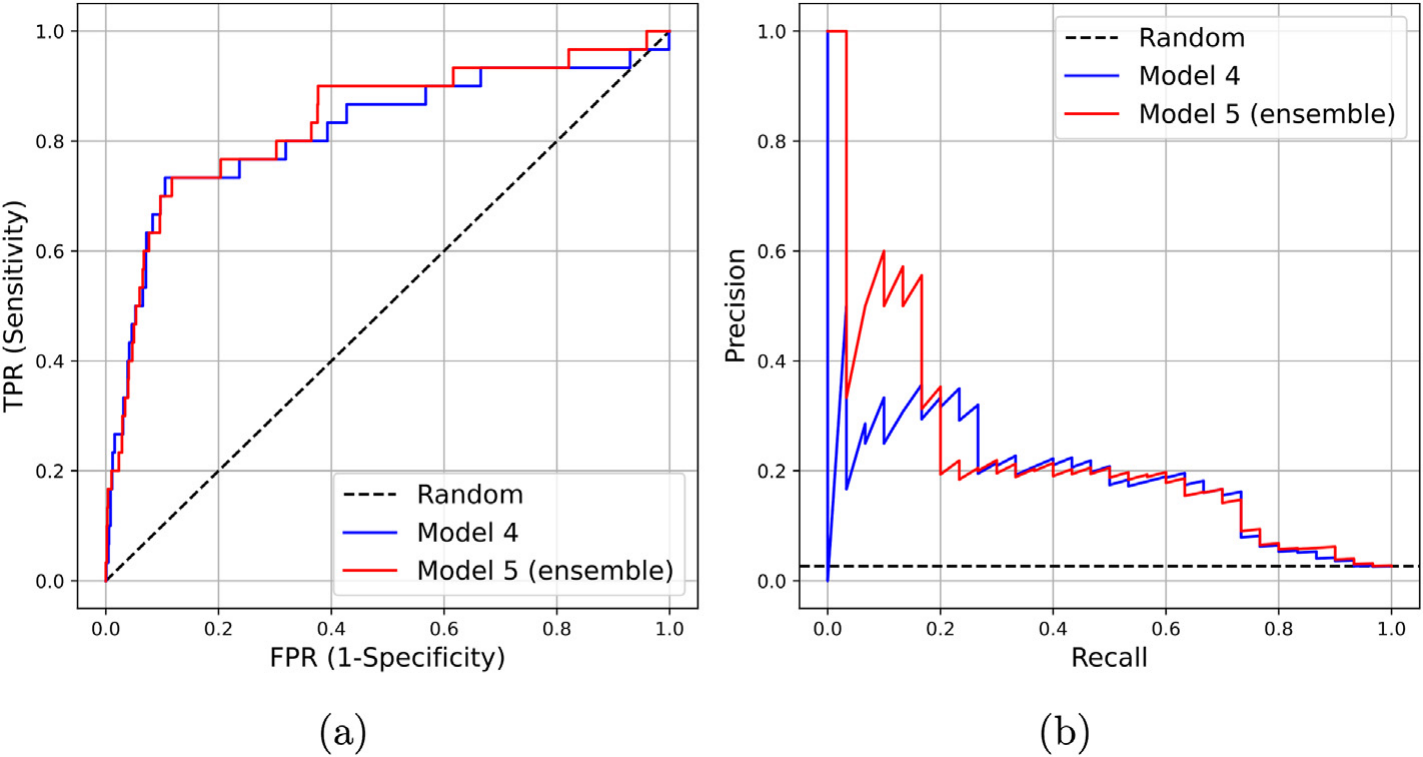
Receiver Operating Curve (ROC) and Average Precision (AP) curves, showing the added value of ultrasonography to the conventional predictive modeling setup that includes patient clinical data, as well as the Kellgren-Lawrence (KL) grade.

The added value of the ensemble model compared to model 4 is graphically shown in Fig. 3. The ensemble model while being less interpretable, yielded a higher AP and AUC than all the models – AP of 0.24 (0.13–0.39) and AUC of 0.83 (0.73–0.91).

### 3.4 Model interpretation

**Fig. 4:**
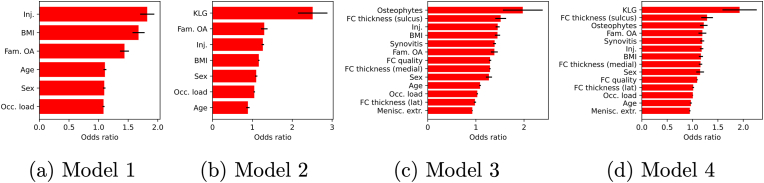
Feature importances (odds ratios) for the model containing patient characteristics, as well as the ultrasound features (model 4 in the text). Black bars indicate standard deviation of importances across leave-one-out evaluations, and the red bars – the corresponding mean values. (For interpretation of the references to colour in this figure legend, the reader is referred to the Web version of this article.)

Fig. 4 shows the feature importance for the models (ORs). The numerical values for these plots are shown in Table 4. Given no other modalities than patient's characteristics, BMI, past injury and familial OA were the main drivers for predicting TKR in model 1. In model 3 (US features and patient-level characteristics), US-detected osteophytes played the biggest role: OR of 2.0 (1.94–2.01). Past injury played a secondary role: OR of 1.5 (1.45–1.46). FCT of the sulcus produced a similar OR of 1.5 (1.51–1.52). BMI OA and presence of synovitis with grade over 1 played the ternary role. The absence of meniscal extrusion was the main factor for predicting the negative class – OR of 0.93 (0.93–0.93). When US examination was combined with the KL grade in model 4 (patient characteristics ​+ ​US features ​+ ​KL grade), the main driver of predicting TKR was the KL grade with OR of 1.9 (1.90–1.96). FCT in sulcus compartment still played the secondary role in explaining TKR predictions and yielded an OR of 1.3 (1.28–1.30). The absence of meniscal extrusion was still the main factor explaining “no-TKR” prediction with OR of 0.95 (0.95–0.95).

## 4 Discussion

In this paper, we have demonstrated that data from ultrasonography allows to predict future total knee replacement. We have shown that while it on itself does not outperform a conventional modeling approach which is based on clinical data and a KL grade, the difference in performance between an ultrasonography model and a KL grade model is rather minor, especially when one considers wider 95% CIs for the KL grade-based model. When combined with radiographic data, ultrasound assessment provides an additional value in TKR prediction, suggesting it has potential as a complimentary tool for clinical practice.

Ultrasound in OA assessment may have multiple clinical advantages as it can be done immediately and, in many occasions, lowering the need for referral to radiography [13] or more costly modalities, such as MRI [34]. In contrast to x-rays it can detect soft tissue changes and meniscal extrusion, which have been reported to be present in the earliest stages of OA and predict later structural changes [35,36].

To our knowledge, this study is the first to make use of ultrasound data and machine learning techniques to predict the future in OA, having several implications. Our work sheds new light on recent findings showing that osteophytes, medial meniscal extrusion, and morphological articular cartilage changes in the medial femoral condyle of the knee joint can be reliably identified by ultrasound [17]. We find about the same prevalence of ultrasound features as in previous studies [16,17]. Here, we show that these data jointly, and when also combined with clinical predictors, can estimate the likelihood of the future total knee replacement. Still, the use of ultrasound in clinical practice will require extensive training in operation and scoring and cannot be immediately implemented as a routine part of the OA joint examination. Future studies may be focused towards automatizing the reading and scoring of ultrasound features during the ultrasound examination, for their immediate use in prediction of future OA outcomes, allowing for an immediate guidance of treatment options.

While our results should be interpreted with some caution because of the low number of cases with total knee joint replacement surgery during follow-up, we emphasize the strength of the evaluation setup in this work. Specifically, in a low-sample setting like ours, it is important to robustly identify hyperparameters of the model (such as e.g. regularization coefficient in logistic regression), and assess the model performance without overfitting. Here, we applied the nested leave-one-out cross-validation procedure that allowed to overcome the biases of model assessment in the small-data regime [37]. Furthermore, we used a simple linear model – logistic regression, to combat overfitting.

It is worth mentioning another methodological strength of this work. Specifically, we used several metrics for the assessment of predictive models – AP and AUC. Here, AP indicates the average positive predictive value and AUC highlights the trade-off between the true and false positive rates. We highlight that the use of AUC as a main metric in the work would be incorrect, since the dataset used for modeling has a very large imbalance of classes (roughly 3% positive cases) [32].

Some important limitations of our study should also be mentioned. First, we could only study persons who reported to be diagnosed with OA in 2010–12 and who attended the clinical examination, with some risk of selection bias. The percentage having knee OA prosthesis at baseline vs. the percentage undergoing joint replacement surgery during follow-up were similar (2.7–3.0%), implying that a portion of participants had end-stage OA already at baseline. However, >80% had baseline Kellgren Lawrence 0–1 and 60% were pain free at baseline, implying that earlier disease stages were widely represented. Hence, our study would have captured any rapid progression.

The second limitation of our work was the amount of missing data on ultrasound. To reflect the real clinical situation as good as possible, we did not do multiple imputations or other actions to minimize consequences of missing data. With more data and less imbalance, we might have been better able to compare the models statistically in terms of non-overlapping confidence intervals of AUC or AP. Third, our analyzes did not consider adjusting for the fact that there were several knees from the same subject. We have, however removed the duplicates from the LOO-CV obtained predictions, and conducted metric evaluation on one knee only, which lifted the scores of our prediction models. Specifically, AP for model 5 was 0.33 (0.21–0.51) and AUC of 0.84 (0.75–0.92). For model 4 – AP was of 0.32 (0.21–0.51) and AUC of 0.82 (0.72–0.91).

The final limitation of this work is that we did not include knee pain as a predictor, although baseline localized and/or widespread pain may be hypothesized to predict future surgery equally well as ultrasonographic and radiographic imaging. This was done to narrow-down the research question and focus on imaging-assessed features. Future work can expand upon this and investigate pain-related questionnaires, quantitative sensory testing and also explore more advanced radiographic assessments, such as quantitative joint space width imaging biomarkers [38].

In conclusion, we found that ultrasound features (osteophytes, meniscal protrusion, suprapatellar synovitis, femoral cartilage thickness and quality) could predict future knee OA surgery cases almost equally well to the Kellgren Lawrence scoring when combined with clinical data. This present work demonstrated the first application of non-traditional OA imaging modality and modeling methodology in prediction of total knee replacement.

## Author contributions

Aleksei Tiulpin and Karin Magnusson had access to all the data in the study and take full responsibility for the integrity of the data and the accuracy of the data analysis. Aleksei Tiulpin performed the statistical analyses. Aleksei Tiulpin and Karin Magnusson drafted the manuscript. Simo Saarakkala, Alexander Mathiessen, Hilde Berner Hammer, Ove Furnes, Lars Nordsletten and Martin Englund, contributed with acquisition of data, conceptual design, analyses and interpretation of results. All authors contributed in drafting the article or critically revising it for important intellectual content. All authors gave final approval for the version to be submitted.

## Role of the funder

This study was funded by the Swedish Research Council (E0234801), the Greta and Johan Kock Foundation, the Swedish Rheumatism Association, the Österlund Foundation, Governmental Funding of Clinical Research within the National Health Service (ALF) and the Faculty of Medicine, Lund University, Sweden. The funding sources had no influence on the design or conduct of the study, the collection, management, analysis, or interpretation of the data, the preparation, review, or approval of the manuscript, or the decision to submit the manuscript for publication.

## Authorship

All authors should have made substantial contributions to all of the following: (1) the conception and design of the study, or acquisition of data, or analysis and interpretation of data, (2) drafting the article or revising it critically for important intellectual content, (3) final approval of the version to be submitted. By signing below each author also verifies that he (she) confirms that neither this manuscript, nor one with substantially similar content, has been submitted, accepted or published elsewhere (except as an abstract). Each manuscript must be accompanied by a declaration of contributions relating to sections (1), (2) and (3) above. This declaration should also name one or more authors who take responsibility for the integrity of the work as a whole, from inception to finished article. These declarations will be included in the published manuscript.

## Acknowledgement of other contributors

All contributors who do not meet the criteria for authorship as defined above should be listed in an acknowledgements section. Examples of those who might be acknowledged include a person who provided purely technical help, writing assistance, or a department chair who provided only general support. Such contributors must give their consent to being named. Authors should disclose whether they had any writing assistance and identify the entity that paid for this assistance.

## Declaration of Funding

All sources of funding should be declared as an acknowledgement at the end of the text.

## Role of the funding source

Authors should declare the role of study sponsors, if any, in the study design, in the collection, analysis and interpretation of data; in the writing of the manuscript; and in the decision to submit the manuscript for publication. If the study sponsors had no such involvement, the authors should state this.

## Studies involving humans or animals

Clinical trials or other experimentation on humans must be in accordance with the ethical standards of the responsible committee on human experimentation (institutional and national) *and* with the Helsinki Declaration of 1975, as revised in 2000. Randomized controlled trials should follow the Consolidated Standards of Reporting Trials (CONSORT) guidelines, and be registered in a public trials registry.

## Declaration of competing interest

All authors have completed the ICMJE uniform disclosure form at www.icmje.org/coi_disclosure.pdf and declare: no support from any organisation for the submitted work; no financial relationships with any organisations that might have an interest in the submitted work in the previous three years; no other relationships or activities that could appear to have influenced the submitted work.
